# Feasibility of Single-lead Cardiac Resynchronization and Defibrillation Therapy in an Animal Model

**DOI:** 10.19102/icrm.2024.15081

**Published:** 2024-08-15

**Authors:** Daniel Varela, Amneet Sandhu, Matthew Zipse, Ryan Gerrard Aleong

**Affiliations:** 1 Cardiology Department, University of Colorado Hospital, Aurora, CO, USA; 2 Cardiology Department, Rocky Mountain Regional VA Medical Center, Aurora, CO, USA

**Keywords:** Cardiac resynchronization therapy, conduction system pacing, single-lead VDD pacing system

## Abstract

Conduction system pacing (CSP) has emerged as an alternative to cardiac resynchronization therapy (CRT); however, there is limited experience with CSP using implantable cardiac defibrillator (ICD) leads. The achievement of CSP with an ICD lead may yield comparable results to cardiac resynchronization therapy defibrillator (CRT-D) therapy using fewer leads. We implanted the Biotronik Linox DX “VDD”-programmable ICD lead in a swine model to investigate the feasibility of “single-lead” CRT-D implantation. With the lead embedded in the basal right ventricular septum, morphologic criteria for CSP were achieved, and successful defibrillation was performed while maintaining atrial sensing. Future work may assure reproducibility of these findings and further determine the feasibility of a single-lead CRT-D.

## Introduction

Conduction system pacing (CSP) has emerged as an alternative to both conventional pacing and cardiac resynchronization therapy (CRT); however, there is limited experience with CSP using implantable cardiac defibrillator (ICD) leads. In patients with heart failure with reduced ejection fraction (HFrEF), the ability to achieve CSP with an ICD lead may allow for resynchronization comparable to CRT, with fewer indwelling leads, less reliance on suitable coronary vein anatomy, and potentially improved procedural efficiency. The Linox DX “VDD”-programmable defibrillator lead (Biotronik, Berlin, Germany) can be used for both CSP and the delivery of tachycardia therapy while maintaining atrioventricular synchrony through its ability to sense in the atrium. As such, the Linox DX lead may serve as a “single-lead” CRT option for select patients with HFrEF.

In this proof-of-concept experiment, we used the Linox DX ICD lead in a well-accepted swine model to investigate the feasibility of “single-lead” CRT-D implantation.

## Methods

This experiment was done with a Yorkshire pig model; the protocol was first approved by the institutional animal care and use committee at our institution (UCHealth University of Colorado Hospital, Aurora, CO, USA).

**Figure 1:**
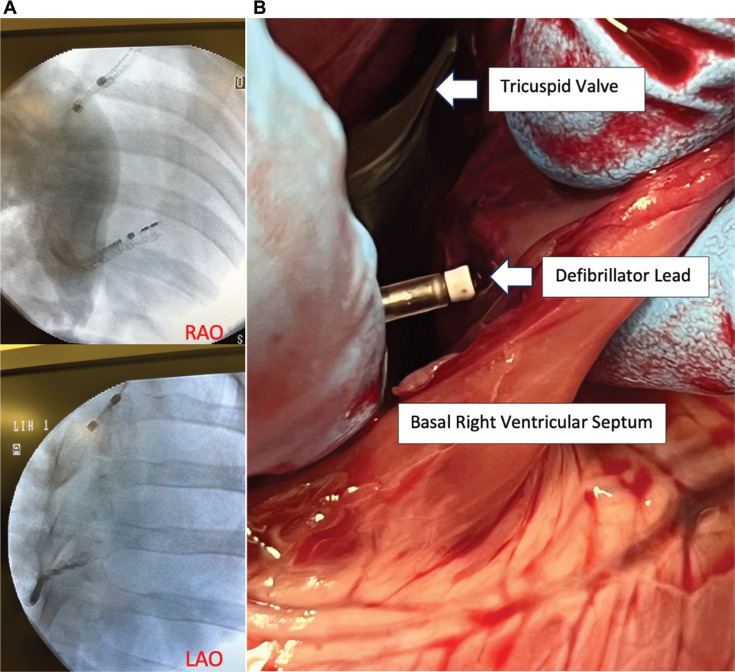
**A:** Right and left anterior oblique views of the Linox DX ICD lead embedded within the basal right ventricular septum. **B:** Pathologic specimen of the dissected pig heart showing the lead’s position with the active fixation screw deployed in the basal right ventricular septum.

A single Yorkshire pig was placed under general anesthesia, and vascular access was obtained in the left internal jugular vein. Using a Worley sheath (Merit Medical, South Jordan, UT, USA), with standard fluoroscopic views and surface echocardiography, the ICD lead was positioned along the basal right ventricle **(Figure 1A)**. Passive lead parameters were recorded, and standard 12-lead electrocardiograms (ECGs) were obtained to demonstrate QRS morphology with conventional basal right ventricular (RV) septal pacing. To obtain CSP, the lead was advanced further into the septum with clockwise rotation of the lead after the screw was deployed, and repeat ECG recordings were obtained to determine QRS morphology and duration as markers for CSP adequacy. With the lead in its final position, embedded within the basal RV septum, lead parameters were once again recorded. A number of parameters have been proposed for the assessment of CSP, including a narrowing of absolute QRS duration, discordance between leads II and III, the development of an r′ or “W” pattern in V1, a left ventricular activation time (LVAT) of <75 ms in V6, shortening of the V6 R-wave peak time (V6 RWPT) by >10 ms when compared to RV septal pacing, an absolute V6 RWPT of <75 ms (in the absence of an underlying conduction system disease), and V6–V1 interpeak interval.^1,2^ Defibrillation testing was then performed with 50-Hz pacing to evaluate for effective defibrillation therapy. The pig was then euthanized, and the heart was explanted to view the position of the lead **(Figure 1B)**.

## Results

**Figure 2:**
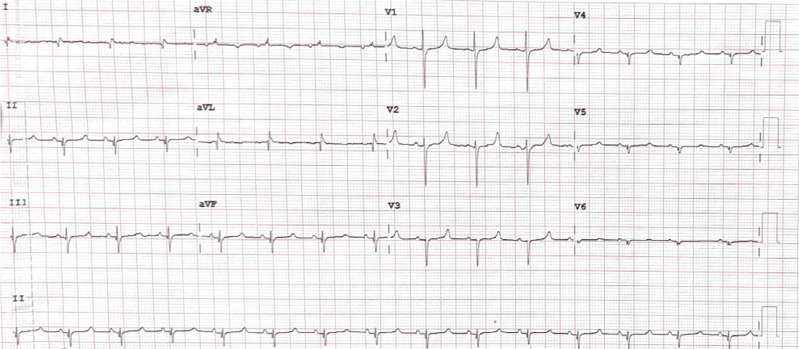
Baseline electrocardiogram of the Yorkshire pig.

**Figure 3:**
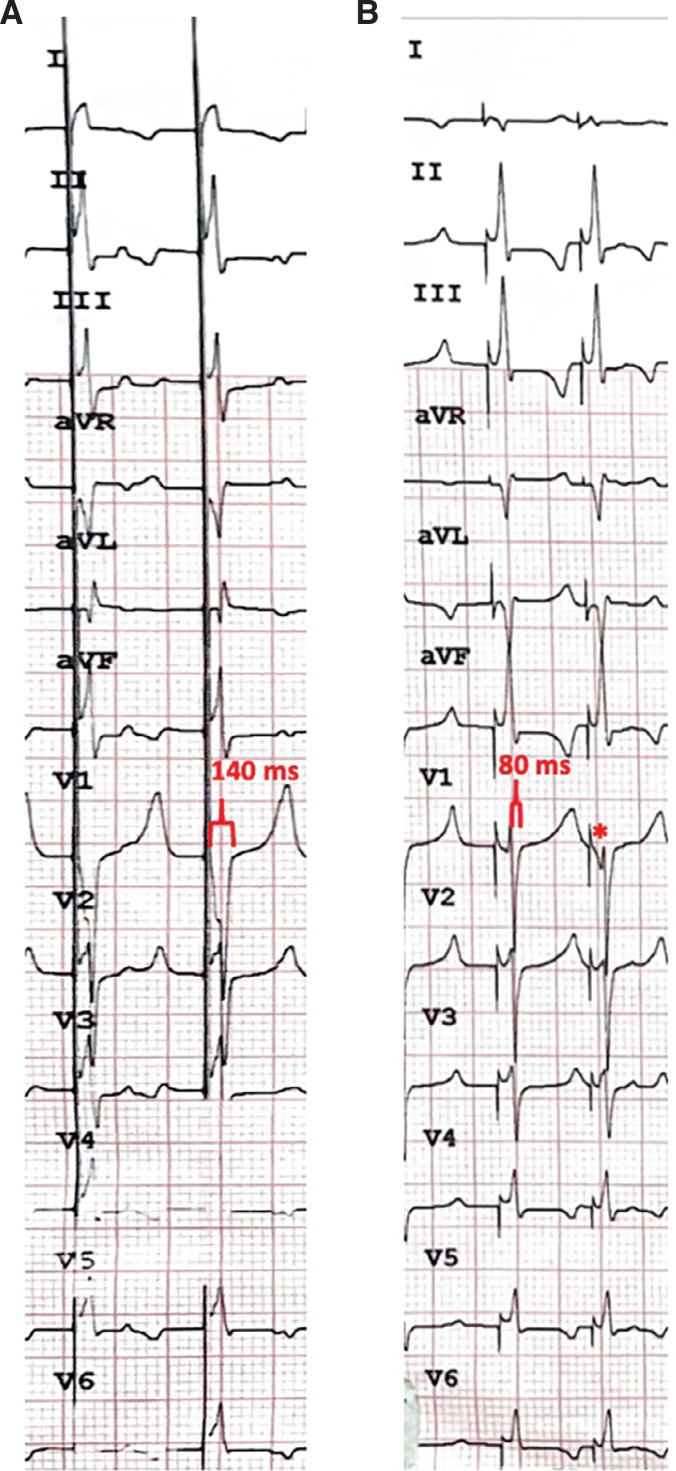
**A:** Conventional basal septal right ventricular–paced morphology with a wide QRS duration of 140 ms. **B:** Conduction system paced morphology with the lead embedded deeper within the right ventricular septum; note QRS duration has narrowed to 80 ms, with an initial Q-wave (*) after the pacing impulse in V1, indicating fascicular capture.

A baseline 12-lead ECG was obtained prior to lead implantation **(Figure 2)**. An ECG analysis during lead deployment revealed a QRS duration of 140 ms and morphology consistent with basal mid-septal RV pacing with the lead in its initial position along the basal septum **(Figure 3A)**. After screwing the lead into the septum, both unipolar and bipolar pacing demonstrated a narrower QRS duration measuring 80 ms, consistent with CSP. Additionally, an initial Q-wave was observed after the pace impulse in V1, indicating engagement of the fascicular system **(Figure 3B)**. While the QRS duration decreased considerably across multiple leads with the ICD lead positioned within the septum, the V6 RWPT was not significantly shortened with CSP and was in fact slightly longer than that observed with RV septal pacing (70 vs. 60 ms).

Lead parameters included sensed P- and R-waves of 4.0 and 12.6 mV, respectively, and a ventricular capture threshold of 0.5 V at 0.4 ms. Impedance was recorded at 1300 Ω, with a shock impedance measuring 68 Ω.

**Figure 4:**
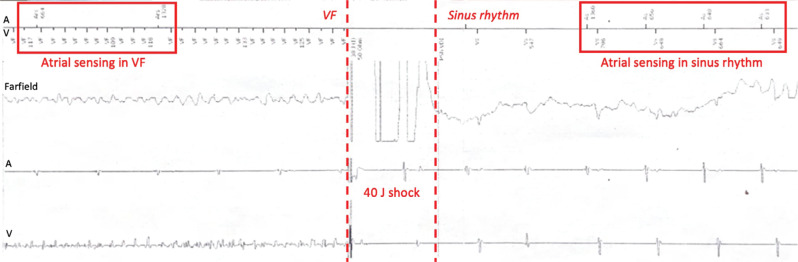
Defibrillation testing at maximum output was successful on the first shock after reversing the shock polarity; atrial sensing was observed in sinus rhythm and preserved during ventricular fibrillation.

Defibrillation testing at maximum output (40 J) was successful after two shocks on the first test and after four shocks on a second test. Shock polarity was then reversed, and a third test was performed, with successful defibrillation achieved with a single shock **(Figure 4)**.

## Discussion

Select patients with HFrEF and left ventricular dyssynchrony have been shown in multiple trials to derive a survival benefit following CRT-D implantation.^3–5^ However, significant heterogeneity lies with the quality of resynchronization, which is highly dependent upon coronary venous anatomy and the presence and location of suitable branches for coronary sinus lead placement. Additionally, CRT-D implantation often requires long procedure times, which may be poorly tolerated among patients with advanced heart failure. CRT-D systems also inherently involve implantation of multiple indwelling transvenous leads, which carry their own host of risks and complications, including infection, vascular stenosis, and tricuspid valvular insufficiency. Due to these concerns, there has been growing interest in CSP via His-bundle pacing and, more recently, left bundle branch area pacing. Liang et al. showed that left bundle branch area pacing allowed for a greater degree of electrical and mechanical resynchronization compared to CRT pacing in patients with non-ischemic cardiomyopathy.^6^ Furthermore, a meta-analysis by Parlavecchio et al. found that, when compared to biventricular pacing, left bundle branch area pacing led to a narrower QRS interval, lower pacing thresholds, and a reduction in heart failure hospitalization rates among patients with HFrEF and left bundle branch block.^7^ While His-bundle and left bundle pacing have already expanded the availability of resynchronization therapies to a larger number of patients, these options may be less ideal options for patients with concurrent indications for defibrillation therapy.

In this initial experiment, we sought to examine the feasibility of a “single-lead” resynchronization–defibrillation device using the Linox DX ICD lead in a well-accepted Yorkshire pig model. We found that morphologic criteria for CSP could be met using standard lead-deployment techniques, primarily evidenced by considerable QRS narrowing. LVAT and V6 RWPT are two other metrics commonly used to assess for CSP adequacy. Despite having not achieved a V6 RWPT of >10 ms shorter than that observed with RV septal pacing, the LVAT and absolute V6 RWPT both remained short (<75 ms), consistent with conduction system capture. There may be additional limitations to using lead V6 alone to determine the adequacy of CSP in our swine model, as the porcine heart sits with a more vertical, and slightly counterclockwise-rotated, orientation in the thoracic cavity, which may alter the QRS morphology and precordial lead vectors relative to that typically seen on an ECG tracing of the human heart.^8,9^ The baseline ECG in our Yorkshire pig demonstrated little to no R-wave in V6, which may limit the utility of metrics that exclusively evaluate this one lead. Another notable difference seen in the conduction system of the porcine heart is the presence of a more robust system of Purkinje–ventricular connections, which results in near-parallel endocardial and epicardial ventricular activation; this anatomic difference may account for the minimal change in V6 RWPT seen with CSP in the swine model, as this metric reflects the time it takes for the depolarization wavefront to reach the epicardial surface of the lateral left ventricle, a process which may still occur quickly even with pure ventricular pacing due to the presence of this more robust system of ventricular–Purkinje connections. Regardless of these anatomic differences, we observed a clear reduction in QRS duration across multiple ECG leads, which may serve as a more accurate assessment for conduction system capture in this swine model. Appropriate lead parameters were also observed in the present study, including adequate atrial sensing, with the lead embedded within the basal RV septum. Despite this non-conventional defibrillation lead position, we demonstrated successful defibrillation, with the ability to discern and treat ventricular arrhythmias at maximum output.

Other investigators have also begun looking into the feasibility of ICD lead implantation in the conduction system. Huybrechts et al.^10^ recently conducted a study on five patients in whom they attempted implantation of a conventional transvenous defibrillation lead into the left bundle branch region and tested the feasibility of achieving left bundle branch pacing morphology and effective defibrillation therapy with the lead in this position. Implantation of the conventional defibrillation lead in the left bundle branch area was reportedly challenging and time-consuming; required the use of multiple sheaths, including those that had to be manually reshaped; and could only be achieved in three of the five patients (two of whom demonstrated left bundle capture, while the other demonstrated left septal capture with pacing). They were able to successfully terminate ventricular fibrillation (VF) during defibrillation testing in all three of these patients. As a feasibility study, the leads were ultimately repositioned in a traditional RV apical position for definitive use in these patients, and long-term data on the position of these leads within the left bundle branch region could not be acquired. Their findings show promise that defibrillation lead implantation in the conduction system may also be feasible in humans, though the challenges the investigators encountered while attempting ICD lead deployment in the left bundle branch area hint to the need for customized ICD leads and delivery sheaths specifically designed for implantation within the conduction system.

### Limitations

The present work represents a feasibility study using a single experimental animal model. As such, additional studies are required to verify the current findings, and no direct comparisons can be made as pertains to the management and care of human patients based on our findings. The evaluation of additional pigs could have allowed for a more comprehensive assessment of all the various markers used to determine the adequacy of CSP across multiple subjects, which would lend further credence to the feasibility of achieving CSP using a defibrillation lead. Also, while we demonstrated that successful defibrillation can be achieved in a swine model at maximum output, additional work is required, with dedicated defibrillation threshold testing, to determine whether successful defibrillation can be achieved at sub-maximal output and to define safety margins for defibrillation therapy. Additionally, while ongoing studies are currently investigating both short- and long-term outcomes for CSP compared to conventional CRT, future studies will also need to investigate these same outcomes using defibrillation leads for CSP, both as standalone single-lead systems or when used in conjunction with other pacing leads. From a procedural standpoint, a specialized long sheath will be needed to deliver an ICD lead in order to achieve CSP. We used a Worley sheath to guide the lead to the septal position, but a specialized sheath designed for CSP for an ICD lead would have been helpful.

## Conclusion

Our findings show that CSP, treatment of ventricular arrhythmias, and atrial sensing could be achieved using a single ICD lead. In this single experiment, we were able to achieve CSP with left bundle branch area pacing and successful defibrillation. Additional research using defibrillation leads for CSP may ultimately lead to the realization of single-lead CRT-D systems, which may serve a unique role in the management of select patients with HFrEF and as indications for resynchronization and defibrillation therapies.
